# How Strong Is Your Coffee? The Influence of Visual Metaphors and Textual Claims on Consumers’ Flavor Perception and Product Evaluation

**DOI:** 10.3389/fpsyg.2018.00053

**Published:** 2018-02-05

**Authors:** Anna Fenko, Roxan de Vries, Thomas van Rompay

**Affiliations:** Department of Communication Science, Faculty of Behavioural, Management and Social Sciences, University of Twente, Enschede, Netherlands

**Keywords:** packaging design, visual metaphor, grounded cognition, textual claim, flavor evaluation

## Abstract

This study investigates the relative impact of textual claims and visual metaphors displayed on the product’s package on consumers’ flavor experience and product evaluation. For consumers, strength is one of the most important sensory attributes of coffee. The 2 × 3 between-subjects experiment (*N* = 123) compared the effects of visual metaphor of strength (an image of a lion located either on top or on the bottom of the package of coffee beans) and the direct textual claim (“extra strong”) on consumers’ responses to coffee, including product expectation, flavor evaluation, strength perception and purchase intention. The results demonstrate that both the textual claim and the visual metaphor can be efficient in communicating the product attribute of strength. The presence of the image positively influenced consumers’ product expectations before tasting. The textual claim increased the perception of strength of coffee and the purchase intention of the product. The location of the image also played an important role in flavor perception and purchase intention. The image located on the bottom of the package increased the perceived strength of coffee and purchase intention of the product compared to the image on top of the package. This result could be interpreted from the perspective of the grounded cognition theory, which suggests that a picture in the lower part of the package would automatically activate the “strong is heavy” metaphor. As heavy objects are usually associated with a position on the ground, this would explain why perceiving a visually heavy package would lead to the experience of a strong coffee. Further research is needed to better understand the relationships between a metaphorical image and its spatial position in food packaging design.

## Introduction

Taste and flavor are the main factors people consider when buying food (Cardello, 1994; Steptoe et al., 1995; Lappalainen et al., 1998). However, in supermarkets and food stores consumers rarely have an opportunity to taste products before purchase. Therefore, when making a purchase decision people rely more on packaging cues and previous knowledge (Creusen and Schoormans, 2005; Schifferstein et al., 2013). Consumers form certain expectations about the taste and flavor of food products by examining different attributes of packaging, such as its color, shape, material, text, typeface and images displayed on the packaging (Deliza et al., 2003; Jaeger, 2006). Expectations affect consumer judgments about food quality and its hedonic properties after tasting and are very robust against later disconfirmation (Cardello and Sawyer, 1992; Tuorila et al., 1994).

The framework of multisensory human–food interaction suggests that sensory attributes of packaging can influence not only the general food expectations, but can directly affect multisensory food experience, such as taste and flavor. Multiple studies within this framework have demonstrated that sensory packaging elements can communicate various attributes of food. For instance, hedonic and health benefits of food products can be successfully communicated by certain combinations of packaging material and color (Fenko et al., 2015b), packaging shape and the sound of product name (Fenko et al., 2016c). Taste intensity and evaluation have been shown to depend on packaging shape (Becker et al., 2011), material (Krishna and Morrin, 2008; Van Rompay et al., 2017) and color (Kauppinen-Räisänen, 2014; also see Spence, 2016 for the overview of the effects of packaging design).

We suggest that not only sensory elements of the package, such as color and material, can influence multisensory food experience, but also the informational elements such as text and images. Most food packaging designs comprise both textual elements (brand name, product category, nutrition information, attribute claims, etc.) and images. Some images are directly related to the contents of the product, depicting a product itself, its ingredients or a meal that can be prepared from it. The images can also be metaphorically associated with the brand and communicate certain brand values (like an image of *Jolly Green Giant* on a pack of canned vegetables or an image of *Pillsbury Doughboy* on a pack of baking powder). Textual claims and images communicate different product properties (e.g., a text can contain a health claim, while an image can communicate hedonic benefits) or the same product attribute (such as intensity, naturalness or freshness).

Metaphorical images are widely used in food packaging, but their effects on consumer responses have not been studied systematically. Furthermore, the relationships between metaphorical images and textual claims that can convey the same message remain unclear. The novelty of our study is determined by its focus on the relative effects of textual claims and metaphorical images on food package and their ability to change multisensory food experience, including flavor perception and product evaluation. The study aims to answer the following research question:

To what extent does a visual metaphor of strength compared to a textual claim of strong coffee depicted on a package of coffee beans influence consumers’ product expectations, flavor perception and purchase intention?

### Textual Claims

Textual claims about sensory and hedonic attributes of food, its ethnic origin, preparation process, various labels (e.g., organic, natural, or health labels) and nutritional information play an important role in consumers’ perception of food and drink (ASAM and Bucklin, 1973; Dubé and Cantin, 2000; Spence and Piqueras-Fiszman, 2014; Piqueras-Fiszman and Spence, 2015). For instance, Wansink et al. (2004) served people a range of savory main courses that had been given either a basic name or a more evocative label (e.g., ‘*Seafood Filet*’ versus ‘*Succulent Italian Seafood Filet*’). The use of descriptive food labels led to a doubling in the number of positive comments from participants, as well as the increase of hedonic ratings of food compared to basic food labels. Similarly, people rated M&Ms as having more intense chocolate taste when labeled as ‘*dark’* rather than as ‘*milk’* chocolate (Shankar et al., 2009).

Textual claims have different effects for specific product categories and specific consumer groups (Lähteenmäki, 2013). For instance, the hedonic label (as opposed to the health label) had a positive effect on consumer responses toward a hedonic product (a chocolate cookie) (Fenko et al., 2016b). The effect was opposite for a healthy product (apple juice), where the health label (as opposed to hedonic label) increased product evaluation and purchase intention. Another study (Fenko et al., 2015a) showed that familiarity and novelty claims can differently affect food neophobic and food neophilic consumers. For neophobic consumers, the familiarity claims positively influenced perceived product familiarity, while for the neophilic consumers, the novelty claims increased taste expectations and purchase intentions.

Most studies agree that textual claims can contribute to a higher perceived attractiveness, quality and purchase intention of products (Machiels and Karnal, 2016). Based on the results of previous findings, we propose the following hypothesis:

H1: A package of coffee beans with a textual claim indicating strong coffee will positively influence product expectations, flavor perception and purchase intention compared to a package without textual claim.

### Visual Images

Imagery on a product’s packaging can have a significant effect on people’s sensory experiences (Bone and France, 2001; Jaeger and MacFie, 2001; Sakai et al., 2005; Cardello, 2007; Mizutani et al., 2010; Liang et al., 2013). For instance, Underwood and Klein (2002) reported that consumers rated products (bacon, margarine, and candy bars) as tasting better when presented with a picture rather than without a picture. Mizutani et al. (2010) have demonstrated that the type of image shown on a drink’s container (whether it is pleasant or unpleasant, and related or unrelated to food) can influence consumers’ perception of hedonic and sensory properties of the orange juice. Deliza et al. (2003) demonstrated that a picture on a product’s packaging can influence consumers’ sensory and hedonic expectations of orange juices. Participants were strongly influenced by the type of picture (either a drawing or a photo) of the product on the package.

In addition to food images, visual imagery may comprise non-food depictions such as brand mascots and background imagery (i.e., a picture of a setting sun or a green field). These images are often used to create a product identity and to promote brand personality (Phillips, 1996). For instance, the *Quaker Oats* logo represents a figure of a Quaker man associated with the values of honesty, integrity and purity. The *Nesquick’s Quicky* bunny evokes instant connotations with speed which implies that consumers can easily prepare the chocolate-flavored drink and quickly drink it. *Cornelius Rooste*r by *Kellogg’s Cornflake*s is a symbol of the early wakening and starting off the day with a healthy breakfast.

### Visual Metaphors

In many cases, usage of visual elements implies metaphor usage whereby the product or brand (target domain) is related to another domain (source domain; see Forceville, 2002). For example, the product attribute of strength (e.g., strength of coffee; target domain) can be metaphorically represented by an image of a strong animal, such as a lion (“*strong as a lion*”; source domain). In consumer research, usage of metaphors has been shown to enhance appreciation of product and brand (McQuarrie and Mick, 2003; Phillips and McQuarrie, 2009).

One reason for this is that metaphors present consumers with a ‘puzzle’ to be solved. For instance, when visualizing a lion’s head on a coffee package to convey strength, consumers have to figure out how ‘*coffee’* (the target domain) and a ‘*lion’* (the source domain) are related. Arguably, solving this ‘puzzle’ is rewarding and hence may inspire positive evaluations (Heckler and Childers, 1992; Ortony, 1993; McQuarrie and Mick, 2003).

We suggest that an image of a strong animal (such as a lion) can serve as a relevant visual metaphor for a strength of coffee. Therefore, we propose the following hypothesis:

H2: A coffee package with an image of a lion as a metaphor for strength will positively influence consumers’ product expectations, flavor perception and purchase intention compared to a package without an image of a lion.

Several studies have shown that explanatory information may enhance visual metaphor effects (e.g., Millis, 2001; Leder et al., 2006), especially when the metaphor is difficult to understand without additional information (Phillips, 2000; Van Rompay and Veltkamp, 2014). Similar idea that verbal texts could support and clarify the implicit meaning of images has been earlier proposed by Barthes (1977) who referred to this phenomenon as “verbal anchoring.” We expect that the effects of metaphor usage will be stronger when accompanied by a textual claim accentuating the metaphor, and thus guiding participants in metaphor ‘resolution.’

Hence, we further propose the following hypothesis:

H3: The positive effects of the metaphorical image of strong coffee on consumers’ product expectations, flavor evaluation and purchase intention will be enhanced when accompanied by a textual claim guiding consumers in interpreting the metaphor.

### Metaphors and Grounded Cognition

Grounded cognition theory argues that so called embodied metaphors represent abstract concepts such as exclusion, power, and intimacy in terms of ‘image-schemas’ (Lakoff and Johnson, 1980, 1999; Van Rompay and Ludden, 2015). For instance, the verticality schema as apparent from utterances such as *‘looking up to someone*’ or ‘*looking down on others.*’ In such expressions, being powerful is linked to looking ‘*down*’ from a position up high, and being weak is associated with ‘*looking up*’ from a position down below (Van Rompay et al., 2005, 2012). Sundar and Noseworthy (2014) found similar effects for the position of brand logos, with a positioning up high generally associated with more powerful brands.

However, depending on the context, verticality may also inspire associations with lightness or heaviness. For instance, Van Rompay et al. (2014) showed that washing powder was experienced as more heavyweight (literally) when visuals were presented on the lower part of the package, a finding in line with earlier research on ‘*heavy’* and ‘*light’* locations in product packaging (e.g., Kahn and Deng, 2010). Furthermore, smell was experienced as lighter and fresher when imagery was presented up high. Jostmann et al. (2009) have demonstrated that people tend to equate heaviness with importance, an association also apparent in language use, for example, “*a weighty issue*” or “*an issue not to be taken light-heartedly.*” Participants that held a heavy clipboard judged monetary value of a product higher that those who held a light clipboard.

Such findings concur with the skepticism people may feel when holding lightweight devices such as a mobile phone. A recent study (Ludden and Van Rompay, 2015) shows that lightweight mobile phones might lower price expectations. Similarly, Fenko et al. (2016a) have demonstrated symbolic congruence between the weight of beer bottles and brand values. Heavy bottles were perceived as representing excellence, authenticity and authority, while light bottles were associated with dynamic and accessible brands.

These studies illustrate the embodied associations between spatial location and sensory constructs related to weight and heaviness, which in the case of coffee might inspire perceptions of a strong taste and aroma. Based on this argumentation, we formulate the following hypothesis (H4):

H4: An image of a lion presented on the bottom of a coffee package will positively influence consumers’ product expectations, flavor perception and purchase intention compared to an image of a lion presented on top of a coffee package.

## Materials and Methods

To look at the relative impact of text and metaphorical images on consumer responses to coffee, we conducted the experiment with the 3 (image on top of the package vs. image on the bottom of the package vs. no image) × 2 (text claim vs. no text claim) between-subjects design. The field experiment took place at one of the Starbucks coffee houses in a medium-sized city in the Netherlands. Participants were exposed to one of the six packaging designs for a fictional brand of coffee beans and were offered a sample cup of coffee allegedly prepared from these beans. The coffee they tasted was a regular coffee from a well-known Dutch brand prepared with a French press and served in a simple plastic cup. Before tasting coffee, participants were asked to look at the package and to evaluate their product expectations. After sampling coffee, participants were asked to evaluate the taste of coffee, its perceived intensity, its expected physiological effects and their purchase intention.

### Participants

To ensure that the experimental sample represented the target population of regular coffee consumers, all participants were recruited among the visitors of a local Starbucks. In total 131 people agreed to participate in the study. All participants were residents of the Netherlands, at least 18 years old, mean age 30 years, 68% were females. A few people who indicated that they did not drink coffee were excluded from the study, resulting in a final number of 123 participants. More than half of the participants (52%) were drinking at least one cup of coffee per day, 33% were drinking a few cups a week, and 10% were drinking just one or two cups a month, and 6% were drinking coffee less than once a year.

Participants characteristics per condition are presented in **Table 1**. A Chi-square test showed that there were no differences in gender distribution between the experimental conditions [χ^2^(5) = 5.29, *p* = 0.38]. A one-way ANOVA confirmed that there were no age differences between conditions [*F*(5,117) = 1.11, *p* = 0.36, non-sign].

**Table 1:** Demographic characteristics of participants per experimental condition.

Condition	*N*	Mean age (*SD*)	Female (%)
Image on top; text claim	20	35.9 (16.2)	60
Image on the bottom; text claim	20	30.9 (15.3)	85
No image; text claim	20	31.3 (14.6)	55
Image on top; no text claim	20	28.3 (13.5)	75
Image on the bottom; no text claim	22	27.7 (11.4)	68.2
No image; no text claim	21	27.1 (13.3)	66.7
Total	123	30.1 (14.1)	68.3

### Stimulus Material

In order to design stimuli for the main study, a focus group pre-study was conducted with professional coffee experts. The participants in the focus group were six baristas from Starbucks, both male and females, between 21 and 29 years old. Participants were asked to come up with a text claim that communicated the product attribute of strong coffee. All participants agreed that the text claim “Extra strong” was the best text claim to communicate the strength of coffee.

Participants were also asked to evaluate 10 different visual metaphors and to select the image most closely associated with the experience of “strong coffee”. The stimuli contained the images of athletes, strong animals (a lion, a wolf, an elephant and a horse) and strong materials (a stone, a rope). The image that received the highest rating was the image of a lion.

**FIGURE 1:**
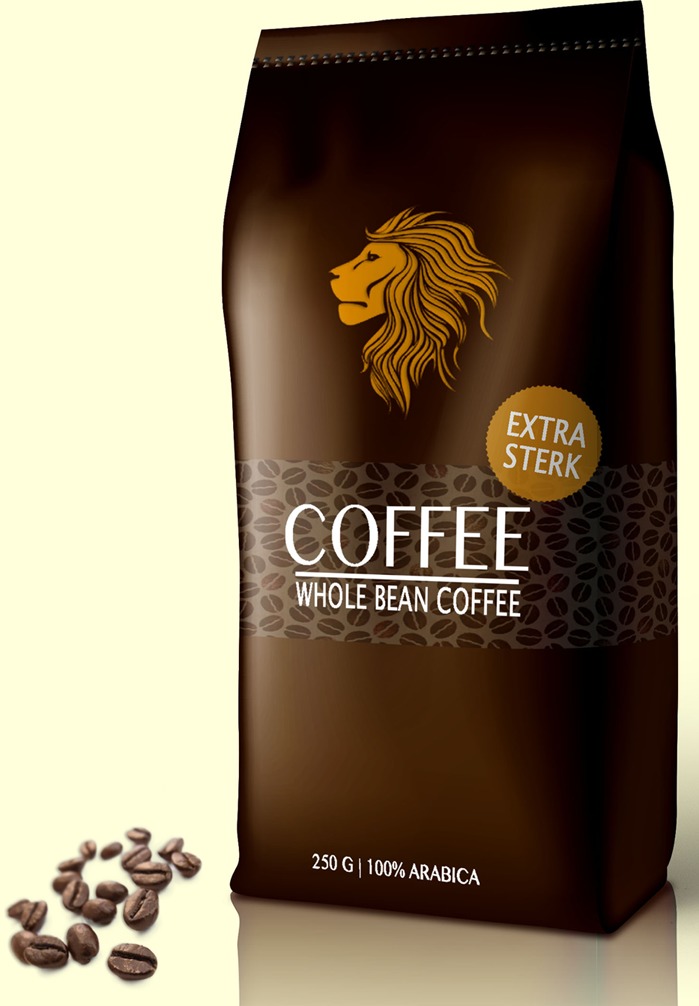
Image on top; the text claim.

**FIGURE 2:**
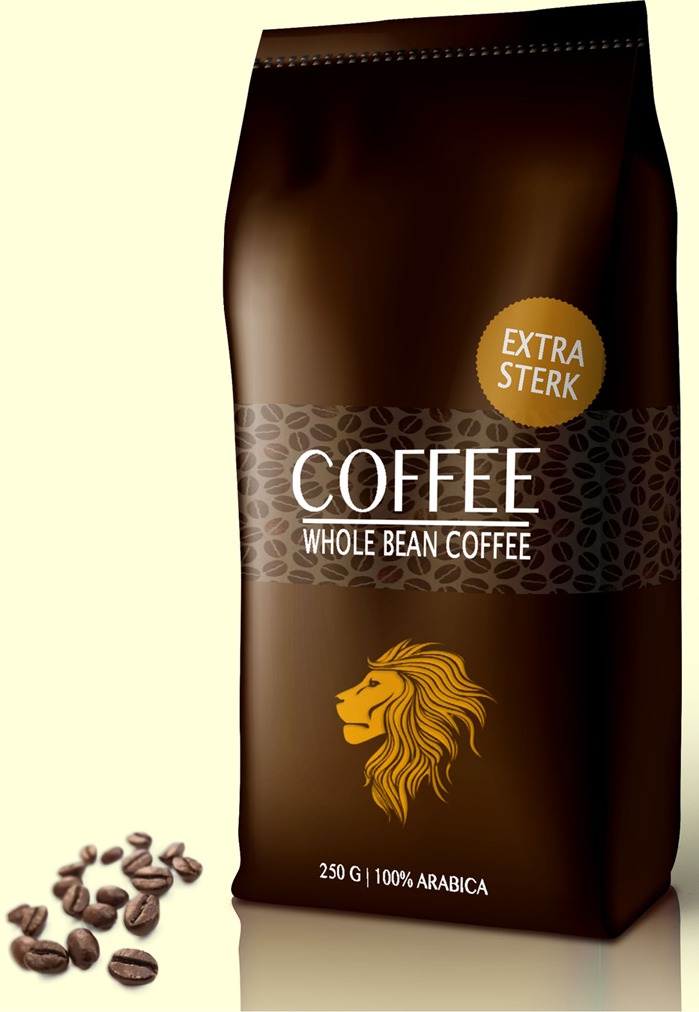
Image on the bottom; the text claim.

**FIGURE 3:**
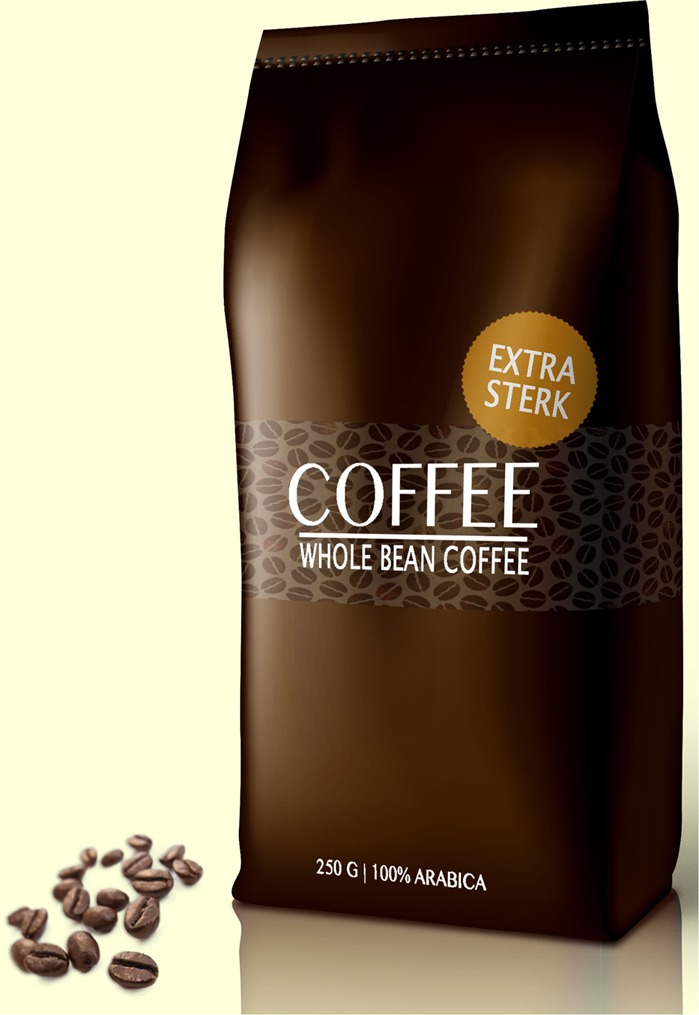
No image; the text claim.

**FIGURE 4:**
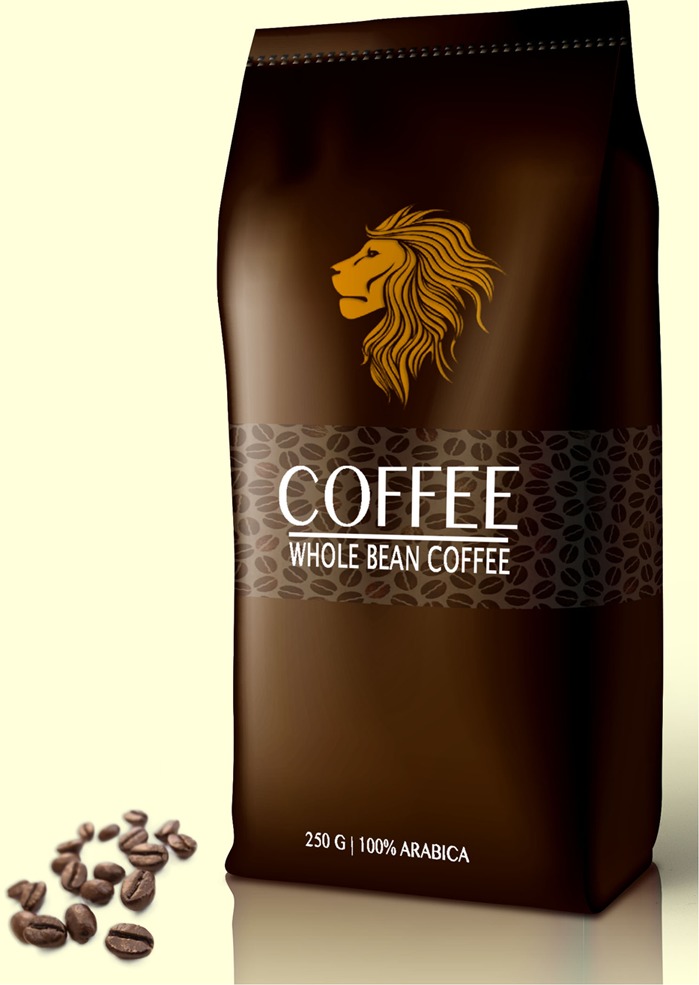
Image on top; no text claim.

**FIGURE 5:**
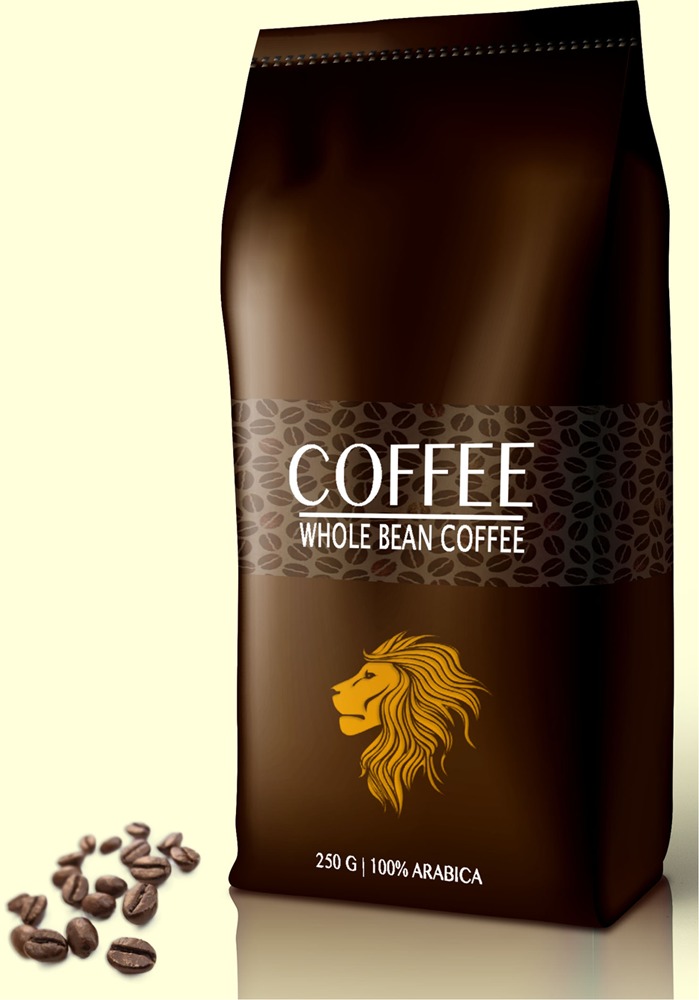
Image on the bottom; no text claim.

**FIGURE 6:**
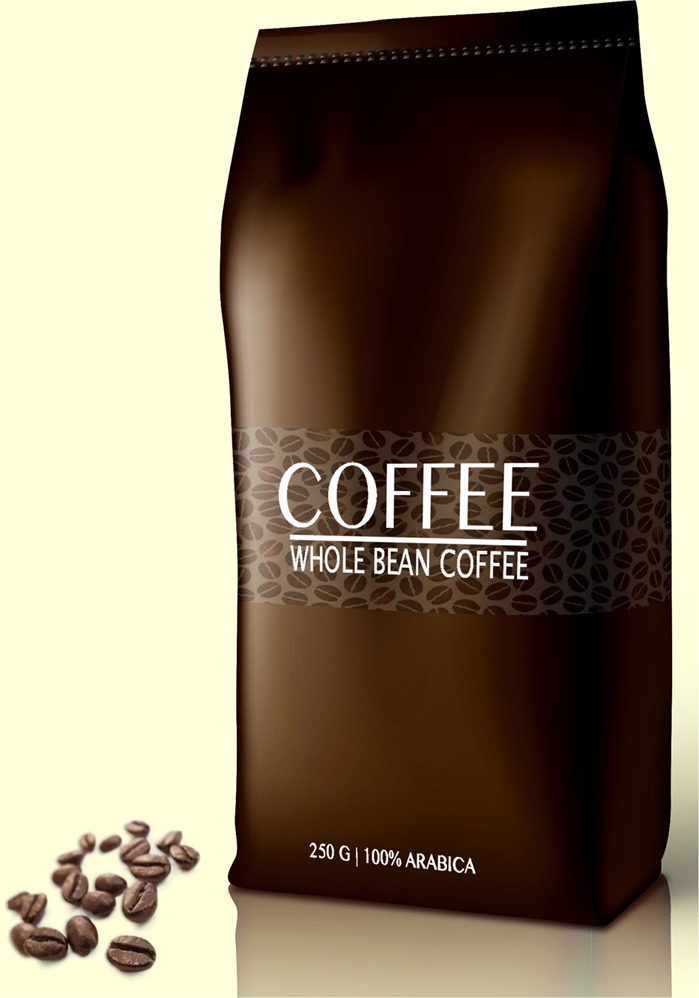
No image, no text claim.

Based on the results of the pre-study, the text claim ‘Extra strong’ (‘*extra sterk*’ in Dutch) and the visual metaphor of a lion were selected for the main study. The final packages for the experimental study are presented in **Figures 1–6**.

The final scales, the items and reliability coefficients are presented in **Table 2**. All items were measured on a 7-point Likert scale from “not at all” to “very.” All scales demonstrated sufficient or high reliability (Cronbach’s α above 0.70, according to Spector, 1991).

**Table 2:** The measurement scales and the reliability of the constructs.

Scale	Items	*N*	Cronbach’s α
Product expectation	‘I like this packaging’	4	0.73
	‘I think this coffee is of good quality’		
	‘This packaging does not appeal to me’ (reversed)		
	‘I think this is a strong coffee’		
Flavor evaluation	‘This coffee tastes good’	4	0.82
	‘This coffee tastes rich’		
	‘This coffee tastes exclusive’		
	‘This coffee tastes cheap’ (reversed)		
Perceived strength	‘This coffee tastes dark’	5	0.90
	‘This coffee tastes heavy’		
	‘This coffee tastes weak’ (reversed)		
	‘This coffee tastes powerful’		
	‘This coffee tastes strong’		
Physiological effects	‘I expect this coffee to make me awake’	7	0.81
	‘I expect this coffee to make me concentrated’		
	‘I expect this coffee to make me focused’		
	‘I expect this coffee to make me lazy’ (reversed)		
	‘I expect this coffee to make me aroused’		
	‘I expect this coffee to make me relaxed’		
	‘I expect this coffee to make me powerful’		
Purchase intention	‘I would consider buying this coffee at the supermarket’	3	0.95
	‘I would buy this coffee at the supermarket’		
	‘There is a strong likelihood that I will buy this coffee at the supermarket’		

### Measures

Recent studies have developed extensive lexicon to describe coffee drinking experience, including long list of emotions (Bhumiratana et al., 2014), pleasantness, emotions and perceptions induced by coffee beverage (Labbe et al., 2015). In our study, we were focused on the more specific sensory experience of “strength” of coffee and hedonic evaluation of flavor. Therefore, we used focus group discussion to extract the most common description of coffee strength and taste evaluation.

Participants in the group discussion agreed that the strength of coffee represents one of its most important attributes. However, coffee experts pointed out that the strength of coffee could be experienced in two different ways: (1) as the intensity of taste and aroma; and (2) as the physiological effects of coffee, such as an increased arousal, energy and alertness. Therefore, two different scales were constructed to measure these two aspects: *Perceived Strength* of coffee and *Physiological Effects* of coffee. When asked to describe physiological effects of coffee, most participants agreed that coffee makes them more awake, energetic, focused, alert and gives them a boost but also a relaxed feeling. This input was used to design a measurement scale for *Physiological Effects* of coffee. Coffee experts were also asked to describe the taste experience of “good” coffee, which resulted in a range of descriptive words, such as sweet, bitter, sour, nutty, smoky and spicy. The descriptions of good or bad coffee differed greatly per person. For instance, “bitter” and “nutty” flavors were evaluated positively by some experts and negatively by others. Therefore, in the final scale only simple descriptions such as “good” or “bad” were used for the *Flavor Evaluation* scale, and a separate scale was constructed for Intensity, or the *Perceived Strength* of coffee.

The final scales of *Product Expectations, Flavor Evaluation* of coffee, *Perceived Strength* of coffee, *Physiological Effects* of drinking coffee, and *Purchase Intention*, the items and reliability coefficients are presented in **Table 2**. All items were measured on point Likert scales from 1 (“Totally disagree”) to 7 (“Totally agree”). All scales demonstrated sufficient or high reliability (Cronbach’s α above 0.70, according to Spector, 1991).

### Procedure

The experiment took place during four working days in April 2017 between 11 am and 5 pm in a Starbucks coffee house in one of the cities in the Netherlands. During the time of the experiment no changes in the environment took place, no additional promotional materials were placed, and the amount of customers remained relatively constant. Customers were approached while they were waiting to order their drinks and were asked to participate in an experiment commissioned by the University of Twente. Participants were guided through the experiment by written instructions. If any of the participants had questions, they were answered by the experimenter. The written instruction to participants contained the following note: “Important: The products that are used in this study have by no means a relation with Starbucks products. The brand Starbucks has no relation with this study.”

The questionnaire was designed in Qualtrics program that was also used to randomized the conditions. Participants were randomly assigned to one of the six conditions. They were shown a package of a fictional brand of coffee beans and were asked to fill out the questions about their *Product Expectations* electronically.

In the second part of the experiment participants were provided with a sample of a coffee supposedly prepared from the contents of the package they had seen. In fact, the coffee they sampled was regular coffee of a popular Dutch coffee brand prepared with a French press and served in a simple plastic cup.

To make sure that the drinks did not differ in sensory properties between participants, the preparation was standardized. Each cup was prepared using 54 g of ground coffee grinded for French press in the Starbucks grinder. The water quality and temperature as well as the time of brewing (4 min) were controlled. The coffee was served to participants immediately after brewing.

After sampling coffee, participants filled out the questions about their *Flavor Evaluation, Perception of Strength, Physiological Effects* of coffee and their *Purchase Intention*. Finally, demographic information and the frequency of participants’ coffee consumption was recorded. The experiment lasted from 5 to 15 min per participant.

## Results

Two-way MANOVA with *Image* (image on top, image on the bottom, or no image) and *Text* (yes or no) as independent factors and *Product Expectations, Flavor Evaluation, Perception of Strength, Physiological Effects* of coffee and *Purchase Intention* as dependent variables was performed. The frequency of coffee consumption was used as the covariate in the model. *Post hoc* tests using the Bonferroni correction were used for post-hoc pairwise comparisons.

**FIGURE 7:**
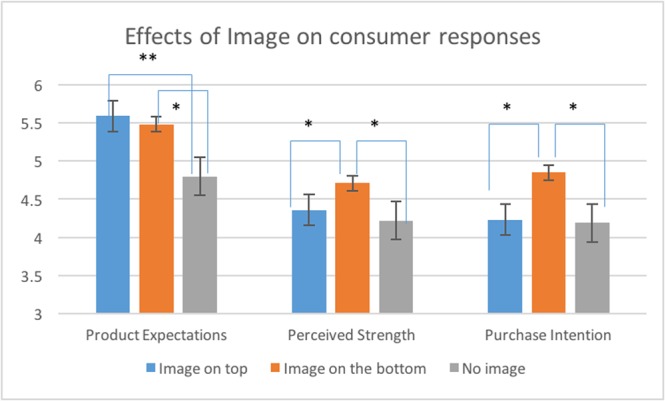
Mean product expectations, perceived strength and purchase intention (±SE) for packages with the image on top of the package, on the bottom of the package and packages without an image. ^∗∗^The effect is significant at 0.01 level; ^∗^the effect is significant at 0.05 level.

The results have demonstrated that *Image* significantly influenced *Product Expectation* [*F*(2,117) = 9.35, *p* < 0.001, η^2^ = 0.14]; *Perceived Strength* of coffee [*F*(2,117) = 4.13, *p* < 0.05, η^2^ = 0.03] and *Purchase Intention* of the product [*F*(2,117) = 5.63, *p* < 0.05, η^2^ = 0.09]. These effects are shown in **Figure 7**. *Post hoc* tests revealed significant differences between both packages with the image and the package without the image (both *p*’s < 0.05). The differences between the images on top and on the bottom of the package were non-significant (*p* > 0.05). Participants had significantly more positive expectations of the product when they saw packages with an image of a lion on top of the package (*M* = 5.59; *SD* = 0.68) or on the bottom of the package (*M* = 5.48; *SD* = 0.69) compared to the package without an image (*M* = 4.80; *SD* = 1.19; *p* < 0.05). However, participants perceived coffee as significantly stronger when they saw the image on the bottom of the package (*M* = 4.71; *SD* = 0.79) compared to image on top (*M* = 4.36; *SD* = 0.96; *p* < 0.05) or the package without an image (*M* = 4.22; *SD* = 1.21; *p* < 0.05). The results for *Purchase Intention* showed a similar pattern: consumers were more likely to buy the product when the image of a lion was positioned on the bottom (*M* = 4.85; *SD* = 0.91) compared to the packages with the image on top (*M* = 4.23; *SD* = 1.00; *p* < 0.05) and no image (*M* = 4.19; *SD* = 1.12; *p* < 0.05). The effects of *Image* on *Flavor Evaluation* and *Physiological Effects* of coffee were non-significant (see **Table 3** for details).

**Table 3:** Main MANOVA results.

Independent factor	Dependent variable	Df	*F*	*P*
Image	Product expectations	2	9.35	<0.01^∗∗^
	Flavor evaluation	2	1.55	0.21
	Perceived strength	2	4.13	0.03^∗^
	Physiological effects	2	1.15	0.32
	Purchase intention	2	5.63	0.04^∗^
Text	Product expectations	1	0.03	0.95
	Flavor evaluation	1	1.29	0.28
	Perceived strength	1	4.42	0.03^∗^
	Physiological effects	1	1.92	0.16
	Purchase intention	1	7.53	0.02^∗^
Image × Text	Product expectations	2	0.21	0.81
	Flavor evaluation	2	0.004	0.99
	Perceived strength	2	0.19	0.83
	Physiological effects	2	0.97	0.37
	Purchase intention	2	1.71	0.18

*^∗∗^The effect is significant at 0.01 level; ^∗^the effect is significant at 0.05 level.*

**FIGURE 8:**
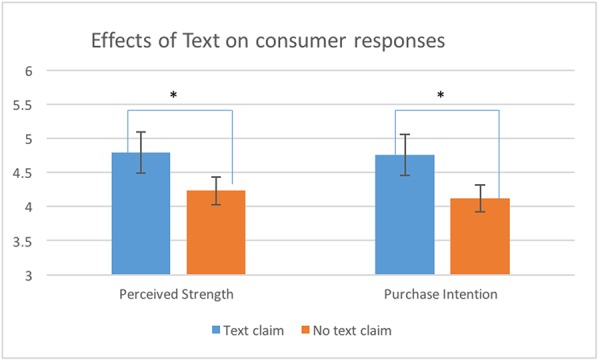
Mean perceived strength and purchase intention (±SE) for packages with and without text claims. ^∗^The effect is significant at 0.05 level.

*Text* significantly influenced *Perceived Strength* of coffee [*F*(1,117) = 4.42, *p* < 0.05, η^2^ = 0.08] and *Purchase Intention* [*F*(1,117) = 7.53, *p* < 0.05, η^2^ = 0.12]. Participants perceived coffee as significantly stronger (*M* = 4.79; *SD* = 1.13) when they saw the textual claim “Extra strong” compared to the package without a claim (*M* = 4.23; *SD* = 0.93). The presence of the claim also significantly increased *Purchase Intention* (*M* = 4.76; *SD* = 1.14) compared to the package without a claim (*M* = 4.12; *SD* = 0.86). Both effects are shown in **Figure 8**. The effects of *Text* on other dependent variables were non-significant. Furthermore, no interaction effects between *Text* and *Image* were found (see **Table 3** for details).

## Discussion

This study was focused on the relative effects of textual claims and metaphorical images presented on the packaging of coffee beans on the consumers’ responses, including product expectations, flavor evaluation, perceived strength and physiological effects of coffee and purchase intention. The results demonstrated that both visual elements and textual claims depicted on the package are able to influence consumers’ responses to the product.

The effects of the textual claim in our study were in line with our first hypothesis (H1). As expected, the textual claim “Extra strong” increased consumers’ perception of the strength of coffee and their intention to purchase the product. However, the textual claim did not seem to influence product expectations, flavor evaluation and the perception of the physiological effects of coffee. These results suggest that a text claim is more efficient in communicating specific product attributes, but not a hedonic experience or general product expectations. A simple and clear message can convince consumers that a product indeed has the attribute claimed and thus result in the higher purchase intention. Therefore, a text claim can be a simple and effective tool for communicating sensory product attributes on food packaging.

In line with our second hypothesis (H2), the presence of the metaphorical image on the package positively influenced consumers’ product expectations before tasting. This result is in line with previous findings that suggested that the attractiveness of images contributes to the evaluation of a product (Jeong, 2008). However, the presence of an image only influenced product expectations, but did not affect actual taste experience and purchase intention.

In our study, no interactions were found between the effects of a textual claim and a metaphorical image. Therefore, our hypothesis that textual claims and metaphorical images can reinforce each other (H3) was not confirmed. These results are in line with the study by Machiels and Karnal (2016) who did not found any interaction effects of text and image on the evaluation of orange juice. Together, these results suggest that images and texts do not merge into an integrated cognitive response, but could be processed in two different systems of information processing (Petty and Cacioppo, 1986; Paivio, 1990; Smith and DeCoster, 2000; Kahneman and Frederick, 2002; Evans, 2008). Textual claims represent informational cues that can be processed rationally. For instance, food labels (such as health, organic or fair trade labels) can evoke consumer skepticism (Tootelian and Ross, 2000; Grunert, 2002; Grunert and Wills, 2007; Sirieix et al., 2013; Fenko et al., 2016b). The fact that consumers express concern about the truthfulness of the information presented on labels and perceive some of the claims as misleading (Szykman et al., 1997; Hamilton et al., 2000; Chan et al., 2005) suggests that textual claims are likely to be processed deliberately and evaluated critically. Images, on the other hand, are perceived in a more holistic way and elicit more emotional responses than textual claims (Messaris, 1997). Pictures are often considered as heuristic cues that lead to peripheral or heuristic processing (Petty et al., 1991). Visual metaphors tend to be implicit and they do not require rational judgment about the credibility of the message (McQuarrie and Mick, 1999; Phillips, 2000). In general, visual images seem to be processed more intuitively and affectively compared to textual claims (McQuarrie and Mick, 1992; Mick, 1992; Peracchio and Meyers-Levy, 1994). Further research is needed to investigate how visual and verbal elements work together and how consumers process textual information and imagery to form integrated responses to products.

In line with our fourth hypothesis (H4), the results demonstrate that the image presented on the bottom of the package positively influenced the perceived strength of coffee and the product’s purchase intention compared to the image presented on top of the package and the package without an image. This result is in line with previous studies that found an association between a lower position of an image on the package and the idea of heaviness (Van Rompay and Veltkamp, 2014). This result can be further explained by the comments made by the participants in our pre-study focus group. They described strong taste as being “heavy” on the tongue. Therefore, our results confirm that the experience of strength can be related to the idea of heaviness and can be therefore communicated by lower spatial position of an image on product packaging.

This result could be interpreted from the perspective of the grounded cognition theory. From this perspective, a picture in the lower part of the package would (automatically) activate the “strong is heavy” metaphor. As heavy objects are in our everyday interactions associated with a position on the ground, this would explain why perceiving a visually heavy package (i.e., with the lion positioned in the bottom part) would lead to the experience of a strong coffee.

It is interesting to note that in our study the product expectations were influenced by the presence of the image, while the perception of strength of coffee and purchase intention are influenced by the position of the image. The reason may be that the expectations are based on the packaging evaluation, which depends on conscious esthetic judgment (how beautiful, how balanced and elegant it looks), while the effects on perceived strength and purchase intention depend on the unconscious, automatic processing of metaphorical meaning, which is reinforced by the lower position of the figure.

Dual information processing (Petty and Cacioppo, 1986; Paivio, 1990; Kahneman and Frederick, 2002) can explain why in certain cases cross-modal correspondences in food experience can be explained by sensory metaphors grounded in embodied experience (such as “warm is kind,” “white is morally pure,” or “strong is heavy”), while in other cases color, shape, image, typeface and other packaging details become “sophisticated form of visual rhetoric” (Scott, 1994) or semiotic codes that can be “decoded” logically by knowledge and historical connotations (Celhay and Remaud, in press).

We believe that in our experiment two types of metaphors were investigated: “strong as a lion” is a conventional metaphor that requires elaborate processing, while “strong is heavy” is a cognitive metaphor that is unconscious by nature. Traditional theories of metaphorical thought suggest conscious and elaborate processing of metaphorical meaning (Mick, 1992). However, grounded cognition theory (Lakoff and Johnson, 1999) challenged this approach, highlighting the systematic patterns underlying metaphorical expressions and their pervasive, mostly unconscious use in everyday language. A rapidly growing body of experimental research have demonstrated the role of cognitive metaphors in human thought (Barsalou, 2008; Williams et al., 2009; Landau et al., 2010). Social and moral concerns tend to be comprehended metaphorically in terms of sensory experiences, such as morality (dirty behavior), sociability (warm person), fairness (even-handed or balanced judgments), importance (weighty matter). These phenomena have been described as “cognitive unconscious”: “It is not merely that we occasionally do not notice these processes”; rather, they are inaccessible to conscious awareness and control (Lakoff and Johnson, 1999, p. 11). Moreover, experiments suggest that metaphorical effects of embodied experiences can be eliminated when people become aware of their nature. For instance, awareness that a weight has been inserted in a book eliminates its metaphorical effect on judgments of the book’s importance (Reinhard et al., 2012).

### Limitations and Suggestions for Future Research

The results of the present study are difficult to generalize, because the study was performed with a single product (coffee beans) and was focused on the single product attribute (strength of coffee). In future research, it is interesting to test whether the results would show similar pattern for a broad range of food products and for different product attributes, including hedonic attributes, healthiness, freshness, naturalness, authenticity, and general quality.

The study also used the limited amount of packaging manipulations. For instance, the visual metaphor was depicted in a rather sketchy manner (only a head of a lion was visible). Previous studies suggest that the style of the visual image (either a drawing or a more realistic photograph) can strongly influence consumers’ sensory and hedonic expectations (Deliza et al., 2003). It is possible that the drawing of an animal’s head alone without a body and the ground on which an animal is standing creates an impression of lightness instead of heaviness. Therefore, it is interesting to test whether the effects of the position will be different if the lion is depicted in full body standing on a high mountain.

The experimental manipulation included only one metaphorical image (a lion) that was congruent with the product attribute (strength of coffee). In the future studies it would be interesting to test whether the effects found in our study could be also found if the image is not metaphorically related to the sensory product property.

Besides, the image of a lion could convey not only a meaning of strong animal suggested by our informants, but also a meaning of royalty (Lion King) or an indication of the origin of coffee beans (an African country). In the future studies on metaphorical images it might be useful to combine quantitative methods (such as scales) with open-ended questions, asking participants directly what kind of meaning the image has to them. Such combination of qualitative and quantitative data might enrich the results of the study and provide more insight into the cognitive mechanisms of metaphorical thinking.

Grounded cognition framework suggests that embodied metaphors are universal (Lakoff and Johnson, 1980). They are grounded in human physical interactions with environment and show little variability across cultures due to universality of bodily constraints (Lakoff and Johnson, 1999). In this study we were focusing at the embodied metaphor grounded in human interactions with objects varying in weight and gravitational forces keeping heavy objects down on the ground. Arguably, such bodily metaphors are stable across cultures compared to culturally informed metaphors.

However, it is also interesting to look at the meaning of visual metaphors from the cross-cultural perspective. Some empirical studies within grounded cognition framework have discovered the effects of culturally specific metaphors. For instance, Lee and Schwarz (2012) found the effect of the smell of fish on adverse effects on cooperative behavior, highlighting the effect of metaphor “fishy is suspicious” which is present only in a limited amount of languages. The meaning of visual metaphors can differ between cultures and even within one culture due to gender and generational differences (Piqueras-Fiszman et al., 2011). In future studies, it is important to take a cross-cultural perspective into account while studying effects of visual metaphors on multisensory food experience.

It is also important to look at the possible moderating variables, such as the product involvement or price sensitivity. Some studies indicate that the response to packaging attributes can depend on consumers’ characteristics, such as design sensitivity (Becker et al., 2011), general health interest (Fenko et al., 2016c), health promotion focus (Karnal et al., 2016) and skepticism toward textual claims (Fenko et al., 2016b). Consumers that are more involved in the product category or specific product attribute (e.g., health benefits) tend to be less sensitive to peripheral cues, such as a packaging shape or the sound of the brand name. It is possible that for these consumers a text claim would provide a more useful information and have a higher influence on the purchase intention. On the other hand, highly involved consumers who consider themselves experts in a product category tend to be more skeptical toward textual claims. Therefore, it is important to further study the influence of text and visual metaphors on consumers with low and high product or attribute involvement.

In our study, no significant effects of either text or image on physiological effects of drinking coffee (arousal) were found. We did not expect to find such effects, because the experiment was too short for each individual participant to notice such an effect. The measure of physiological effects was included in order to separate two meanings of “strengths” of coffee that we had found in the pre-study focus group. Asking separate questions about the strengths of taste and the expected strengths of arousal allowed us to measure the effect of packaging on flavor perception (“the strengths of taste”) more precisely. However, in future studies it would be interesting to test the effects of packaging information (textual and pictorial) on physiological arousal measured both subjectively and by using objective methods such as hart rate and skin conductivity.

## Conclusion

This study demonstrates that the use of textual claims and metaphorical images depicted on a package of coffee beans can significantly change consumers’ product expectations, the strength perception of coffee and purchase intention. Textual claims seem to be more efficient in directly communicating clear and simple product attributes, such as strength and thus positively influence purchase intention. A metaphorical image depicted on the package is able to communicate the same attribute in an indirect way. In addition, an image can enhance the esthetic quality of the package and increase general product expectations. The location of the image on the package is also important for consumer experience. The results of our study suggest that placing an image on the bottom of the package can metaphorically communicate certain product attributes (such as intensity, heaviness and strength) and thus increase consumers’ purchase intention for consumers who value these attributes.

## Ethics Statement

This study was carried out in accordance with the recommendations of Ethical Committee of the Faculty of Behavioural Science of the University of Twente. All subjects gave written informed consent in accordance with the Declaration of Helsinki. The protocol was approved by the Ethical Committee of the Faculty of Behavioural Science of the University of Twente (COMMISSIE ETHIEK (CE) FACULTEIT GEDRAGSWETENSCHAPPEN; protocol # 17077).

## Author Contributions

All authors listed have made a substantial, direct and intellectual contribution to the work, and approved it for publication.

## Conflict of Interest Statement

The authors declare that the research was conducted in the absence of any commercial or financial relationships that could be construed as a potential conflict of interest.
